# Fabrication and Stability Improvement of Monoglyceride Oleogel/Polyglycerol Polyricinoleate-Stabilized W/O High Internal Phase Pickering Emulsions

**DOI:** 10.3390/foods13121944

**Published:** 2024-06-20

**Authors:** Yingzhu Zhang, Jinqi Xu, Jinhua Gong, Yan Li

**Affiliations:** 1 College of Food Science and Technology, Huazhong Agricultural University, Wuhan 430070, China; yzzhang0925@163.com (Y.Z.); xu15007168135@163.com (J.X.); gjh15833867830@163.com (J.G.); 2 Key Laboratory of Environment Correlative Dietology, Huazhong Agricultural University, Ministry of Education, Wuhan 430070, China

**Keywords:** freeze–thaw stability, texture, plasticity, internal phase composition, creams

## Abstract

To decrease the lipid content in water-in-oil (W/O) emulsions, high internal phase Pickering W/O emulsions (HIPPE) were fabricated using magnetic stirring using a combination of monoglyceride (MAG) oleogel and polyglycerol polyacrylate oleate (PGPR) as stabilizers. Effects of MAGs (glyceryl monostearate-GMS, glycerol monolaurate-GML and glycerol monocaprylate-GMC) and internal phase components on the formation and properties of HIPPEs were investigated. The results showed that milky-white stabilized W/O HIPPE with up to 85 wt% aqueous phase content was successfully prepared, and the droplet interfaces presented a network of MAG crystals, independent of the MAG type. All HIPPEs exhibited great stability under freeze–thaw cycles but were less plastic. Meanwhile, GML-oleogel-based HIPPEs had larger particle size and were less thermal stable than GMS and GMC-based HIPPEs. Compared to guar gum, the internal phase components of sodium chloride and sucrose were more effective in reducing the particle size of HIPPEs, improving their stability and plasticity, and stabilizing them during 100-day storage. HIPPEs presented great spreadability, ductility and plasticity after whipping treatment. This knowledge provides a new perspective on the use of oleogels as co-stabilizers for the formation of W/O HIPPEs, which can be used as a potential substitute for creams.

## 1. Introduction

High internal phase emulsions (HIPEs), typically comprising over 74% of an internal phase, belong to a type of highly viscoelastic concentrated emulsion. Their excellent characteristics, such as solid properties, controllable consistency and high internal fraction, make them a desirable medium for low-fat product manufacturing and bioactive compound encapsulation [1,2]. High internal phase Pickering emulsions (HIPPEs) are formed by adsorption of solid colloidal particles at the oil–water interface of the HIPEs, which determines the formation of a solid network structure that promotes the stabilization of oil/water droplets [3]. Compared with traditional emulsifiers, the solid particles form thick and porous shell structure at the oil–water interface, which improves the anti-aggregation and stability of the emulsion [4,5]. Although research on inorganic particle stabilizers has been widely reported [6,7], researchers are increasingly turning their attention to fat-, protein- and polysaccharide-based particle stabilizers in the pursuit of “clean-label” foods [8,9].

Compared to oil-in-water (O/W) emulsions, water-in-oil (W/O) emulsions with water as the internal phase have a variety of potential applications in the production of low-fat foods and fat substitutes. Margarine and butter are representative of foodstuffs in water-in-oil Pickering emulsion systems, which are driven by the stabilization of Pickering crystals at the oil–water interface and the establishment of a network of continuous-phase lipid or fat crystals [10]. Fat crystals as Pickering particles are firmly anchored to the droplet surface, creating a steric barrier to effectively prevent the droplet coalescence [10,11]. Nonetheless, W/O HIPEs still belong to a class of kinetically unstable systems. High concentration of the internal phase increases droplet aggregation and water separation, leading to instability of the system. Therefore, enhancing the stability of W/O HIPEs remains an urgent issue for the food industry.

Polyglycerol polyricinoleate (PGPR) is a hydrophobic and semi-synthetic emulsifier which has traditionally been employed to stabilize W/O HIPEs. However, the use of PGPR with high concentration in food products is often limited. To minimize reliance on PGPR and enhance the edibility and stability of W/O HIPEs, the food industry has explored the use of oleogelaters based on particle crystallization, including monoglyceride (MAG), diacylglycerol (DAG) and beeswax to address this challenge [12,13]. As a special emulsifier and oleogelator, MAG exhibits excellent ability in stabilizing oleogel-based W/O HIPEs by forming strong crystal networks with desirable structural properties. By controlling the chain length and type of MAG, the crystallization behavior and physical properties of MAG-based oleogels can be regulated, thereby determining the performance of oleogel-based W/O emulsion systems. Liu et al. utilized the synergistic effect of DAG and PGPR to prepare W/O HIPE, and the results showed that the addition of DAG had a positive effect on improving the stability of HIPE [14]. When DAG concentration was 6 wt%, only 1 wt% PGPR was needed to co-stabilize HIPEs with up to 80% water content. Lee et al. enhanced the stability and rheology of W/O HIPEs by incorporating beeswax and glycerol monooleate (GMO) into the oil phase [15]. W/O HIPEs were formulated based on oleogel and hydrogel by selecting GMO as the interface stabilizer and beeswax to establish a structured network within the external phase, leading to the improved performance. Moreover, the incorporation of structural agents into the internal aqueous phase is a promising approach to improve the stability of W/O HIPEs. Adding electrolyte compounds such as NaCl to the inner phase of PGPR/DAG-stabilized W/O emulsions could increase the electrostatic repulsive force between water droplets, promote the adsorption of PGPR and reduce the interfacial tension of the stabilizers, thus improving the stability and mechanical properties of the emulsions [14]. In addition, hydrophilic colloids including gellan gum and carrageenan can be used as stabilizers and structural agents within the aqueous phase. The gelling and thickening properties of hydrocolloids can enhance water droplet anti-condensation, thereby increasing the strength of W/O emulsions [12]. In most reports of oleogel-stabilized emulsions, the formation of fat crystals was achieved by cooling the prepared emulsion, which may alter the natural crystalline properties of fat. Our previous work reported that the formation and properties of oleogels depend on the type of MAG [16]. Therefore, we aimed to use oleogel directly as a co-stabilizer to stabilize HIPPE along with PGPR without additional cooling treatment.

Therefore, MAG-based oleogel and PGPR were used as the stabilizer to prepare W/O HIPPEs by low-energy emulsification method without further cooling treatment. The influence of different MAG-based oleogels on the formation and freeze–thaw stability of HIPPEs was evaluated. Subsequently, guar gum, NaCl and sucrose were selected as the internal phase components and their effects on the storage stability and textural properties of HIPPEs were investigated. Finally, the effect of whipping treatment as one of the food processing procedures was investigated.

## 2. Materials and Methods

### 2.1. Materials

Glyceryl monostearate (GMS, analytical grade), glycerol monolaurate (GML, purity ≥ 90%) and glycerol monocaprylate (GMC, analytical grade) were purchased from Yuanye Biotechnology Co., Ltd. (Shanghai, China). Polyglycerol polyricinoleate (PGPR, food grade) was obtained from Dahe Food Technology Co., Ltd. (Zhengzhou, China). Sucrose (food grade) was provided by Jiangxi Qiaosao Food Co., Ltd. (Zhangshu, China). Nile red was provided by Sigma-Aldrich Co. (St. Louis, MO, USA). Soybean oil and commercial cream were purchased from a local market in Wuhan, China. All other chemicals used were of analytical grade.

### 2.2. Preparation of Oleogels and HIPPEs

Oleogels: Oleogels were prepared according to our previous report [16]. In detail, each MAG was precisely weighed and added into soybean oil at a constant concentration of 6 wt%. The mixtures were heated to 80 °C in a water bath and stirred at 500 rpm until the MAG was thoroughly dissolved in soybean oil. Subsequently, the oil solutions were quickly cooled to room temperature (~25 °C) in ice bath and then stored at 4 °C for 24 h to obtain oleogels for further use.

HIPPEs: The preparation of HIPPEs was conducted according to the formulation in Table 1. The oil phase was prepared by adding a certain amount of PGPR into pre-prepared oleogels. Distilled water was slowly added into the oil phase under constant stirring at 500–800 rpm for 10–20 min to obtain W/O HIPPPEs with water phase proportion of 75–90 wt% and PGPR proportion in oil phase of 0.5–2 wt%. Without PGPR, W/O emulsion could not be formed when water phase proportion was higher than 70 wt%. Due to the low fluidity of oleogels, the stirring speed and mixing time were controlled and varied to make sure the formation of W/O HIPPEs. To study the effect of internal phase components, different concentrations of sucrose (5–15 wt%), NaCl (0.5–1.5 wt%) or guar gum (0.01–0.1 wt%) were incorporated into the water phase of HIPPEs with an internal phase proportion of 85 wt% (*w*/*w*). The corresponding emulsions were prepared according to the above steps. All the HIPPEs were named as MAG-oleogel HIPPEs.

In order to identify W/O and O/W emulsions, a straightforward procedure was applied [17]. To be specific, a drop of emulsion was dropped into two separate vessels with pure water and with pure oil, respectively. Then the miscibility of emulsion in water and oil was observed, and it was found that W/O emulsions could be well dispersed in pure oil, while kept insoluble and drops in pure water. For O/W emulsions, the phenomenon in oil and in water was the opposite. 

### 2.3. Freeze–Thaw Treatment

Each emulsion sample with approximately 50 mL was transferred to anti-freezing glass containers and frozen in a refrigerator at −18 °C for 24 h. The samples were subsequently thawed in a water bath at 25 °C for 2 h and then subjected to continuous stirring at 500–800 rpm for 10–20 min to re-generate the emulsions. This procedure was repeated for a total of four freeze–thaw cycles. For reference, GMS oleogel HIPPEs with 85%, 80% and 75% aqueous phase were labeled as S-1, S-2 and S-3, respectively and the corresponding HIPPEs after freeze–thaw cycling were labeled as FT-S-1, FT-S-2 and FT-S-3, respectively. For GMC and GML, C- and L- were used to label samples instead of S- for GMS.

### 2.4. Whipping Treatment

In order to test the plasticity of HIPPEs, a hand-held automatic stirrer (DDQ-B01K1, Bear, Foshan, China) was used. Fresh emulsion (200 g) was accurately weighed into a stainless whipping bowl and then whipped by the stirrer. The power of the stirrer was set at 125 W and the whipping time was around 8 min for the formation of soft spikes [18].

### 2.5. Microstructure Observation

The microstructure and crystal structure of samples were visualized using a polarized light microscope (MG-100, M-shot, Guangzhou, China) equipped with a digital camera. An appropriate quantity of the emulsion sample was gently applied to the microscope glass slide and a coverslip was placed on top to ensure homogeneity. The microstructure was observed at 25 °C using a 20× objective lens operated in both bright field and polarized light modes.

The distribution of oil droplets in the freshly prepared and freeze-thawed samples was observed by using an inverted fluorescence microscope (TI-S, Nikon, Tokyo, Japan). Nile red dye was prepared at a concentration of 0.01% (*w*/*w*) by dissolving it in anhydrous ethanol. A tiny amount of the sample was evenly spread on the microscope glass slide and 5 μL of nile red solution was added dropwise for staining. A coverslip was covered above and then positioned under a fluorescence microscope with a 20× objective lens to capture fluorescence images.

### 2.6. Particle Size Measurement

Image analysis software (ImageJ 1.53e, National Institutes of Health, Bethesda, MD, USA) was used to measure over 60 droplets in each microscopic image to estimate the size of the emulsion droplets. The average droplet size was calculated directly by using the ImageJ software.

### 2.7. Rheological Characterization

To assess the rheological behavior of samples, a rheometer (DHR2, TA, New Castle, DE, USA) equipped with a Peltier plate thermostat was employed. A 40 mm diameter aluminum parallel plate was selected, and the geometry gap was kept at 1000 μm. Stain sweep tests were first conducted at a frequency of 1 Hz and a stress range of 0.001–100% to investigate the linear viscoelastic region (LVR) of the samples. Subsequently, frequency sweep tests were carried out in a range of 0.1–100 Hz with a constant strain of 0.1% and the viscosity changes were measured alternately at a shear rate of 0.1 s^−1^ and 10 s^−1^ to evaluate the thixotropic recovery ability of the samples. All the above testing procedures were measured at 25 °C. Finally, temperature sweep tests were performed at 1 Hz frequency and 0.1% strain at a 2 °C/min rate. This allowed for exploration into the effect of temperature on the rheological characteristics of the samples, through two sweeps consisting of heating and cooling processes within a range of 5–60 °C.

### 2.8. Transverse Relaxation Analysis

Approximately 1.0 g of emulsion sample was weighed and placed in a special tube for nuclear magnetic resonance detection (NMR) by using an NMR imaging analyzer (NMI20-015V-I, Niumag, Suzhou, China). The CPMG sequence was selected, and the test parameters were set as follows: time data TD = 2,700,052, sampling bandwidth SW = 100 KHz, number of echoes NECH = 18,000, number sampling = 8, echo time TE = 1.5 ms, time wait TW = 8000 ms, data radius DR = 1, spectrometer frequency SF = 21 MHz. The T2 values were fitted using the T2-Fit 4.0 software after the scan test. All procedures were performed at room temperature (25 °C).

### 2.9. Storage Stability

The freshly prepared emulsions were collected into the bottles and then inverted. All the filled bottles were placed at 4 °C for 100 days. The visual images were taken at 0-, 14-, 30- and 100-day.

### 2.10. Shape Retention Ability of Whipped Samples

In order to assess the short-term shape retention ability of the samples, the whipped samples were analyzed photographically. Horizontal shaped wreath samples and three-dimensional shaped spike samples were produced using a star-shaped decorative head, and then placed on a level surface at 25 °C. The visual images were taken after 0, 1, 3, 5 and 24 h storage.

### 2.11. Interfacial Tension

The oil–water interfacial tension was measured at 25 °C using an interfacial rheometer (Tracker Teclis, IT Concept, Lyon, France). A pendant drop module-a hook needle was used to create a pendant drop of water. The pendant drop was formed by injecting the aqueous phase into a quartz cell containing around 3/4 of the oil phase, through an automatic injection unit. The oil phase consisted of soybean oil and its mixture with various concentrations of MAG and/or PGPR. To obtain the time-dependent interfacial tension curves, a computer simulation of the Young–Laplace equation was conducted.

### 2.12. Water Holding Capacity

Water holding capacity (WHC) of HIPPEs was determined according to the method by Han et al. [19]. In brief, approximately 20 g of freshly prepared emulsion samples were placed in a 50 mL centrifuge tube and centrifuged at 10,000 rpm for 20 min using a frozen centrifuge (H1850R, Cence, Changsha, China). After centrifugation, the lower aqueous phase was extracted using a syringe and the solid phase was weighted. To determine WHC, the mass of the centrifuge tube was first recorded as “a” (g), the total mass of the sample and the centrifuge tube before centrifugation was noted as “b” (g), and the mass after removal of the water phase post-centrifugation was recorded as “c” (g). The WHC was calculated using the following equation:

(1) WHC (%)=(c−a)/(b−a) × 100

### 2.13. Hardness

The hardness of the emulsion samples was determined using TA. XT Plus texture analyzer (Stable Micro Systems, London, UK). A P/45 C probe was chosen, and the test parameters were set as follows: a pre-test speed of 2 mm/s, a test speed of 1 mm/s, a post-test speed of 5 mm/s, a trigger force of 3 g and a puncture distance of 20 mm. The maximum force during the TA test was considered as the hardness value.

### 2.14. Statistical Analysis

All the experiments were repeated at least thrice for each sample, and the resulting data were expressed as mean +/− standard deviation. One-way ANOVA was applied to analyze the significant differences between samples using SPSS version 25.0 software.

## 3. Results and Discussion

### 3.1. Effect of MAG-Oleogels and PGPR on the Formation of W/O HIPPEs

**Figure 1:**
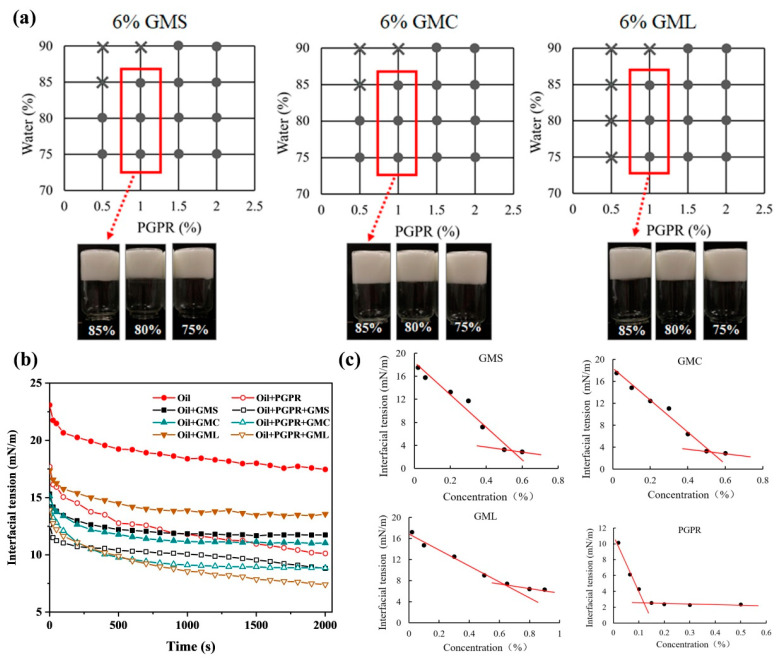
Phase diagram and the visual appearance of the MAG–oleogel–based W/O HIPPEs (**a**). Curves of interfacial tension versus time (**b**) and Interfacial tension balance values at different concentrations of GMS, GMC, GML and PGPR in soybean oil (**c**). (×) denotes oil–water separation mixture, (●) denotes the formation of HIPPEs. All the measurements were carried out under 25 °C.

**Figure 2:**
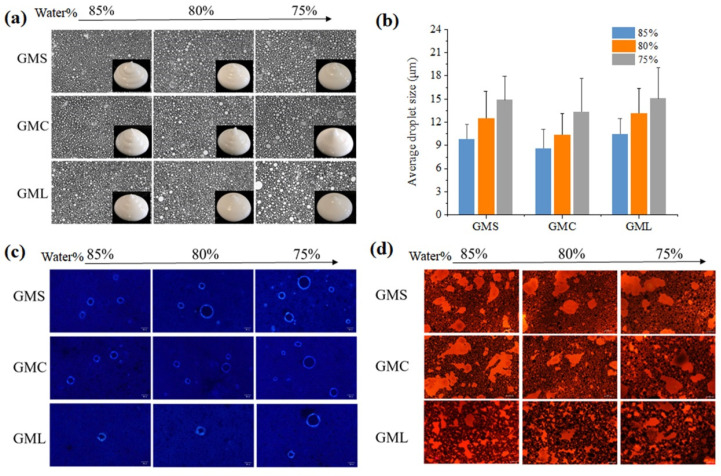
Optical micrograph and sample images (**a**), average droplet size (**b**), polarized light microscopy images (**c**) and fluorescence images (**d**) of MAG–oleogel–based W/O HIPPEs. The scale bar is 50 μm.

Magnetic stirring as the low-energy homogenization method was used in the present work. All the formed emulsions in the phase diagram were identified as W/O type due to their uniform dispersion in pure oil while aggregating in pure water. MAG-oleogels or PGPR alone were unable to form HIPPEs, which could be formed in the combination of MAG-oleogel and PGPR. Hence, the formation of MAG-oleogel/PGPR-based W/O HIPPEs were optimized in Figure 1a. At ≥85 wt% water phase content, three MAG-oleogels in a combination of 0.5 wt% PGPR failed to form a stable emulsion, resulting in oil–water separation. When lowering the content of water phase (75–80%), GMS- and GMC-oleogel could stabilize emulsions in the presence of 0.5 wt% PGPR, rather than GML-oleogel. When PGPR content was 1% and higher, all the emulsions were formed with water phase of 75–85 wt%, but not for 90 wt%. The visual appearance of W/O HIPPEs stabilized by MAG-oleogel and 1 wt% PGPR were solid-like in the bottle. The emulsions exhibited creamy texture, smooth surface, milky white and moderate plasticity (Figure 2a, inset). There were no apparent differences in the appearance of the three MAG-oleogel-based HIPPEs under varying water phase proportions (75–85%). The results confirmed that the synergistic effects between MAG-oleogel and PGPR facilitated the formation of W/O emulsion, in consistence with the findings of Liu et al. [13,18].

The synergistic effects between MAG and PGPR were further determined by the measurement of interfacial tension between oil and water. The interfacial tension between soybean oil and water decreased from 23.08 mN/m to 17.46 mN/m within 2000 s (Figure 1b), which may be caused by a minority of emulsifying components such as phospholipids and fatty acid glycerides in the oil phase. The presence of PGPR, GMS, GMC and GML in the soybean oil effectively decreased the interfacial tension to 10.12 mN/m, 11.74 mN/m, 11.03 mN/m and 13.56 mN/m, respectively. Figure 1c presented that PGPR had the lowest CMC of 0.12%, and CMC of GMS, GMC and GML were 0.55%, 0.53% and 0.65%, respectively. The results of interfacial tension and CMC indicated that PGPR had better emulsifying ability than MAGs. PGPR has been widely used to stabilize W/O emulsions, due to excellent amphiphilicity and emulsifying ability [20,21]. In contrast, the interfacial tension further decreased to 8.82 mN/m, 8.86 mN/m and 7.41 mN/m for samples containing PGPR/GMS, PGPR/GMC and PGPR/GML, respectively, confirming a synergistic effect between PGPR and MAGs. The emulsification ability of MAGs was mainly attributed to the presence of hydrophobic fatty acids and hydrophilic hydroxyl groups in the MAG molecules [22]. According to the above results, oleogels formed by 6% MAG and 1% PGPR were combined to form W/O HIPPEs in the following studies.

### 3.2. Effect of MAGs and Water Phase Content on the Properties of HIPPEs

All the obtained HIPPEs were milky white and viscous, and water droplets inside were uniformly distributed and packed together (Figure 2a). The droplet size of HIPPEs varied from 7 μm–20 μm, slightly decreasing with higher water content (Figure 2b). A previous study by Ojeda-Serna et al. also found that the smaller particle size of the emulsion with increasing water phase content [23], which might be related to the limited amount of MAG at the oil–water interface [24,25]. The morphology and location of MAG crystals and oil phase was examined using polarized light microscopy and fluorescent microscopy (Figure 2c,d). Figure 2c displayed that fat crystal shell was observed around the droplets and in the continuous phase, confirming the surface activity of MAG and MAG-oleogels and benefiting for the physical stability of emulsions [10]. With the water content increased, the fat crystal shell gradually decreased, revealing that under higher water content, the gelator incorporated in the W/O emulsion was less adsorbed at the interface and primarily distributed in the continuous phase [23]. Under fluorescent microscopy, both oil phase distributed around the water droplets and oil aggregation were observed (Figure 2d), further proving that both the adsorbed MAG and lipid crystals in the interface contributed to the stabilization of HIPPEs [18]. MAG types had no significant effect on the formation and microstructure of HIPPEs.

**Figure 3:**
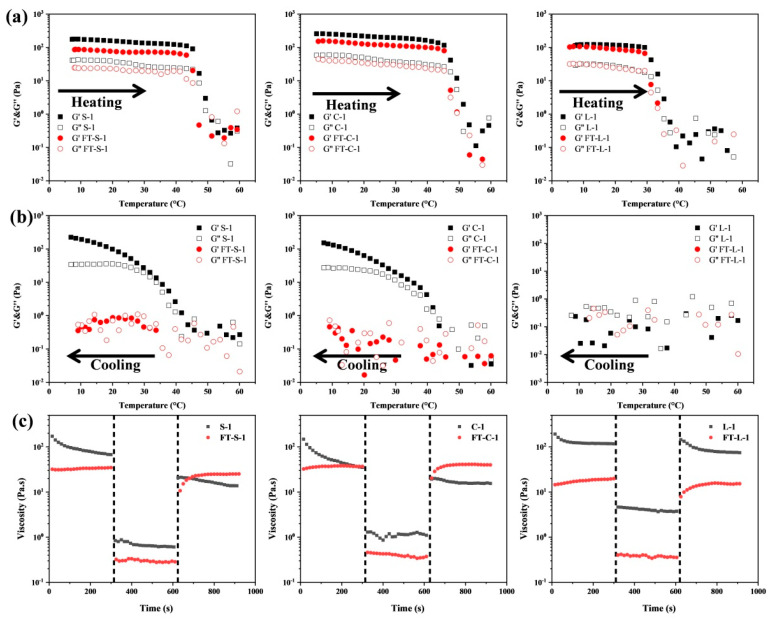
Temperature sweep curves during heating (**a**) and cooling (**b**) process and the thixotropic properties (**c**) of MAG–oleogel–based W/O HIPPEs (φ = 85%) before and after four freeze–thaw cycles.

The rheological properties of HIPPEs were first analyzed by using a strain-scan test (Figure S1a–c). All the emulsions had a wider LVR range of 0.001–10%, where the modulus of HIPPEs kept stable with the increasing oscillation strain. When deviating from LVR, the structure of HIPPEs would be damaged due to the higher oscillation strain, causing the decrease of modulus. G’ of all samples was greater than G”, indicating the stable and viscoelastic properties of HIPPEs. A similar phenomenon was reported in soy protein isolate emulsion gels [26] and whey protein emulsion gels [27]. The results also showed that modulus values (G’ around 100 Pa) of GMS- and GMC-oleogel HIPPEs were higher than those of GML-oleogel HIPPEs (G’ less than 100 Pa) under the same aqueous phase content (Figure S1a–c and Figure 3), implying a stronger gel network of GMS- and GMC-oleogel HIPPEs. It might be due to the higher hardness of GMS- and GMC-oleogel than GML-oleogel [16]. But water content had less effect on the rheological properties of HIPPEs (Figure S1 and Figure 3).

Due to the presence of MAG, all the HIPPEs were thermal sensitive, independent on water content (Figure 3a,b). Taking HIPPEs with a water content of 85% as an example, during heating, both G’ and G” of HIPPEs kept stable, which then underwent a rapid decline when temperature was higher than a critical value, indicating the structure breakdown of HIPPEs. HIPPEs containing GMS- and GMC-oleogel had a melting point of approximately 45 °C, while those containing GML-oleogel had a slightly lower melting point of approximately 30 °C. Due to the high thermal resistance of GMS- and GMC-oleogels, GML had lower crystallization capacity than GMS and GMC at low temperature [16]. As a result, HIPPEs containing GML-oleogel could not re-form the gelled structure during cooling test, causing little or no change in modulus. Figure 3c further depicted the thixotropy of HIPPEs, which is important for food products like cream, mayonnaise where a reversible structure breakdown and recovery during shearing and storage are required. The thixotropic recovery of viscosity depended on MAG types and water content.

### 3.3. Effect of Freeze–Thaw Treatment on the Structure and Properties of HIPPEs

**Figure 4:**
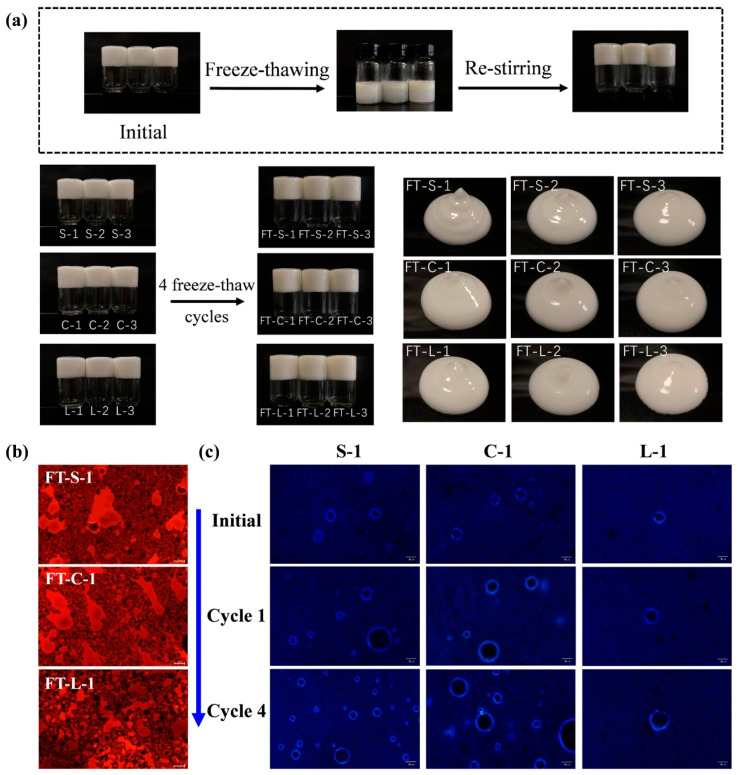
The schematic diagram of the freeze–thaw cycle treatment and the visual appearance of the oleogel-based W/O HIPEs after four freeze–thaw cycles (**a**), fluorescence images (**b**) and the polarized light microscopy images (**c**) of the oleogel-based W/O HIPEs (φ = 85%) after freeze–thaw cycles. The scale bar is 50 μm.

The freeze–thaw cycle treatment is common in food processing, which might negatively affect food quality. As illustrated in Figure 4a, phase separation occurred in the thawed emulsion after freezing, with a solid oil phase in the upper layer and an aqueous phase in the lower layer. In the frozen state, ice crystals formed in the emulsions. Upon thawing, the interconnected frozen droplets melted and coalesced, and water could penetrate from the interface, thus separating the water phase [10]. It is noteworthy that stable emulsions re-formed under agitation due to the use of a low-energy homogenization method for emulsion preparation (Figure 4a). After four freeze–thaw cycles, the appearance of the emulsion was restored after re-mixing.

The distribution of oil and water phase before and after freeze–thaw treatment was presented in Figure 4b,c, which was in line with the results in Figure 2c,d. However, after freeze–thaw cycles, more droplets with lipid crystal shells were observed in HIPPEs containing GMS- and GMC-oleogels, while no change in HIPPEs containing GML-oleogel (Figure 4c). This meant that the stirring force applied to the HIPPEs during the freeze–thaw cycle promoted the absorption of GMS and GMC at the oil–water interface. GML crystals were fibrous with larger size, which might be difficult to diffuse and absorb at the interface [16]. Overall, the freeze–thaw treatments had no adverse effect on the microstructure of emulsions, indicating the combination of PGPR and MAG-oleogels benefited the stability of emulsions during freeze–thaw cycles. Hence, freeze–thaw treatment had no significant effect on the rheological and thixotropic recovery properties of HIPPEs (Figure 3 and Figure S1). Also, water distribution in HIPPEs kept no change before and after freeze–thaw treatment (Figure S1a′–c′). The above results revealed that HIPPEs co-stabilized by oleogel and PGPR showed high freeze–thaw stability.

### 3.4. Influence of Internal Phase Composition on the Storage Stability and Properties of HIPPEs

**Figure 5:**
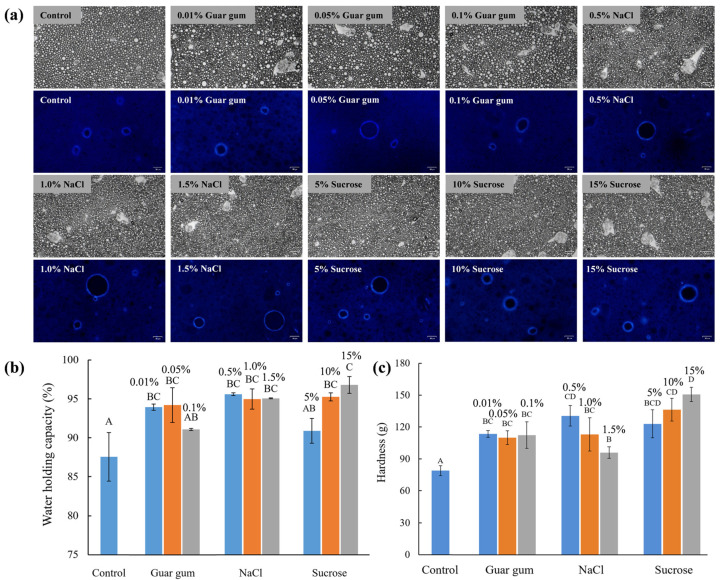
Optical micrograph and sample images and polarized light microscopy images (**a**), water holding capacity (**b**), the hardness (**c**) of GMS–oleogel–based W/O HIPPEs in the presence of internal phase components (guar gum, NaCl and sucrose). The scale bar is 50 μm. Different capital letters indicate significant differences among different samples, *p* < 0.05.

**Figure 6:**
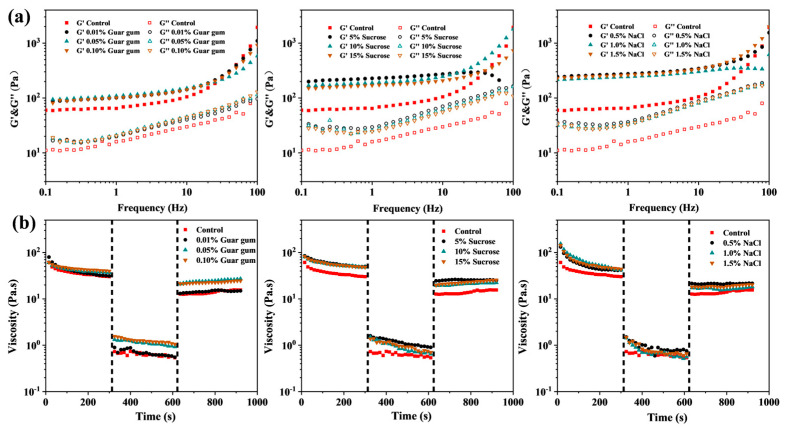
Dynamic frequency sweep curves (**a**) and thixotropic properties (**b**) of GMS–oleogel–based W/O HIPPEs (φ = 85%) with different internal phase composition.

Although MAG-oleogel-based HIPPEs had good freeze–thaw reversibility, they were less stable during 4 °C storage with phase separation occurring after 14 days (Figure S2). It was reported that some compounds, such as NaCl and gallic acid, could be used to enhance the stability of W/O emulsions [20]. HIPPE based on GMS-oleogel (85 wt% water content) was then used as a matrix and guar gum, sodium chloride and sucrose were added to its internal aqueous phase. The addition of those components showed no effect on the appearance and microstructure of the emulsions but increased their solidity, especially in the presence of NaCl and sucrose (Figure 5), resulting in great improvement on the storage stability (Figure S3). In the presence of 0.05% and 0.1% guar gum, HIPPE showed structural collapse after 30 and 100 days of storage, respectively. However, with the addition of NaCl and sucrose, HIPPE remained stable during 100 days of storage. Meanwhile, water holding capacity and hardness of HIPPEs also increased in the presence of those internal phase components (Figure 5b,c). Wolf et al. observed that NaCl addition generally increased the stabilization performance of PGPR [28]. But when NaCl concentration was relatively high, altering NaCl concentration yielded no significant difference in particle size. Hence, the improvement on the storage stability of HIPPEs by addition of different components might be due to other physical properties. Figure 6a presented that the addition of sucrose, NaCl and guar gum in the internal aqueous phase significantly increased the G’ and G” values of HIPPEs, but independent of the concentration of those fillings. G’ of HIPPEs containing NaCl and sucrose was more than 100 Pa, while that of HIPPEs containing guar gum was less than 100 Pa. The addition of internal phase components affected thixotropic recovery ability of HIPPEs regarding sucrose, NaCl and guar gum (Figure 6b).

The stabilizing effect of internal additives such as salt and sugar on W/O emulsions may be achieved by reducing the droplet size and the attraction between water droplets, and increasing the adsorption density of emulsifiers [29]. Raviadaran et al. also elucidated that the addition of NaCl in the aqueous phase reduced the average particle size of W/O emulsion droplets [21]. The results indicated that NaCl, as an ionic compound, could be dissociated into Na^+^ and Cl^−^ in water, which facilitated the formation of electrostatic repulsion, thereby reducing attraction between water droplets and contributing to a reduction in the average particle size of the emulsion. NaCl addition was reported to promote the interaction between the hydrophobic chains of PGPR, and the cations could be used as the bridge to connect the hydrophilic polyglycerol chains, thereby increasing the rheology of the interfacial film [29]. Moreover, the decrease of emulsion droplet size would increase both the surface area of the interaction between droplets and the friction between adjacent droplets, thereby enhancing the viscosity of W/O emulsions [20]. Sucrose is reported to increase the viscosity and density of emulsion systems and reduce the activity and migration of water, thus modulating the hydrophobic interaction among stabilizers and affecting the solvent polarity [30,31]. Hence, sucrose might affect the stability and properties of emulsion-based systems [32,33,34]. As thickening and gelling agents, hydrophilic colloids (e.g., κ-carrageenan) could enhance the standing ability of W/O HIPEs when presented in their internal phase [14,15]. Guar gum is a water-soluble and gel-forming galactomannan, whose thickening properties helped the emulsion stability against coalescence and separation [35]. Therefore, the improvement of storage stability of HIPPE depended on the content of guar gum in the internal phase (Figure S2). However, when the content of guar gum was greater than 0.1 wt%, it was difficult to encapsulate in the internal phase of HIPPE due to its high hydrophilicity. In short, the incorporation of sucrose, NaCl and guar gum in the aqueous phase can be used to modulate the rheological properties of HIPPEs and thus improve their stability.

### 3.5. Influence of Whipping Treatment on Texture and Properties of HIPPEs

**Figure 7:**
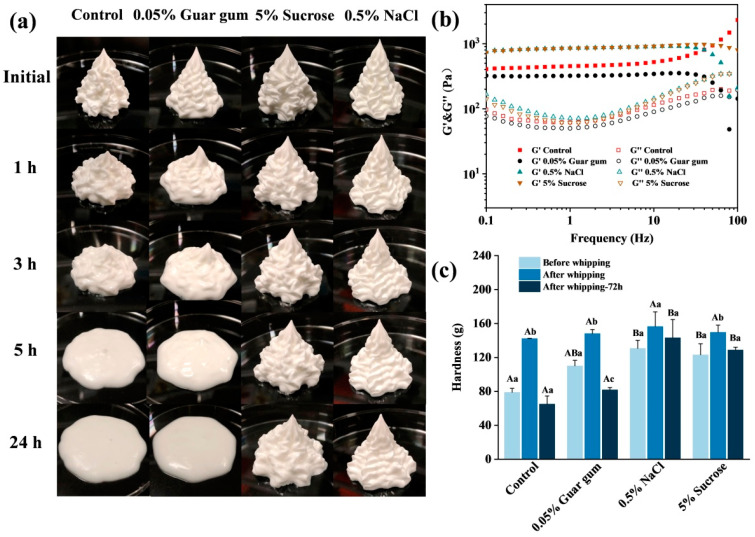
Effect of whipping treatment on the short-term storage stability (**a**), rheological properties of dynamic frequency sweep curves (**b**) and hardness (**c**) of GMS–oleogel–based W/O HIPPEs (φ = 85%) with different internal phase composition. Different capital letters represent significant differences in samples with different internal additives under the same treatment conditions (*p* < 0.05). Different lowercase letters represent significant differences in samples with the same internal additives under different treatment conditions (*p* < 0.05).

Whipping treatment was used to test the plasticity of GMS-oleogel-based HIPPEs with the addition of 5% sucrose, 0.5% NaCl or 0.05% guar gum. As shown in Figure 7a and Figure S4a, HIPPEs could be shaped with good plasticity via whipping treatment. However, the duration of shape maintenance was different for each formulation. The shape of GMS-oleogel-based HIPPE destroyed after 3 h storage and became fluidic after 5 h, similar to HIPPE containing 0.05% guar gum, which resulted in the reduction of hardness (Figure 7c). In contrast, sucrose and NaCl addition helped to maintain the shape of HIPPEs for 24 h. The results might be due to higher modulus and hardness in the presence of NaCl and sucrose than guar gum (Figure 7b,c). The plasticity of HIPPEs containing NaCl and sucrose was similar to that of the commercial animal and plant creams (Figure S4). Droplets in both HIPPEs and commercial cream samples were stabilized by fat crystals and network, but those in plant cream had larger particle size. The above findings indicated that HIPPEs had good plascitity which could withstand whipping treatment.

## 4. Conclusions

W/O HIPPEs were directly produced using MAG-oleogels and PGPR as the co-stabilizers. After optimized 1 wt% PGPR and 6 wt% MAG level, water content in HIPPEs could reach up to 85%. The type of MAG had slight effect on the appearance and microstructure of HIPPEs. All the HIPPEs exhibited excellent reversible freeze–thaw and whipping stability. While the rheological properties of GML-oleogel-based HIPPEs became worse during cooling treatment than HIPPEs containing GMS and GMC-oleogels. The storage stability of HIPPEs greatly enhanced by the addition of guar gum, NaCl and sucrose, which might be due to the improvement of the water holding capacity, mechanical property and viscoelasticity. NaCl and sucrose were more effective for regulating the textural properties of HIPPEs than 0.05 wt% guar gum by increasing their spreadability, plasticity and rigidity after whipping treatment. GMS-oleogel-stabilized W/O HIPPEs with 85% water phase and containing 5 wt% sucrose or 0.5 wt% NaCl within the internal phase exhibited the most desirable mechanical properties. But the specific improvement mechanism needs to be elucidated. The present HIPPEs based on MAG-oleogels has excellent mechanical properties and can be potentially used for a wide range of products such as creams, low-fat foods, fat substitutes and delivery systems.

## Figures and Tables

**Table 1:** Formulation optimization and texture improvement of HIPPEs and experimental design.

Formulation Optimization of HIPPEs		Texture Improvement of GMS-Oleogel-Based HIPPEs with 85% Water	
Type of MAG (Oleogelator)	Water Content	Internal Phase Composition	Content
GMS	75%, 80%, 85%	Guar gum	0.01%, 0.05%, 0.1%
GMC	75%, 80%, 85%	NaCl	0.5%, 1.0%, 1.5%
GML	75%, 80%, 85%	Sucrose	5%, 10%, 15%
Variants of experimental design		Variants of experimental design	
Microstructure observation, droplet size, rheological characterization, freeze–thaw treatment		Microstructure observation, droplet size, rheological characterization, freeze–thaw treatment	

## Data Availability

The original contributions presented in the study are included in the article and Supplementary Materials, further inquiries can be directed to the corresponding author.

## Supplementary Materials

The following supporting information can be downloaded at: https://www.mdpi.com/article/10.3390/foods13121944/s1, Figure S1: Strain sweep curves of GMS (a), GMC (b) and GML (c) oleogel-based W/O HIPPEs before and after freeze–thaw cycles. Water distribution of GMS (a′), GMC (b′) and GML (c′) oleogel-based W/O HIPPEs before and after freeze–thaw cycles; Figure S2: Temperature sweep curves during heating (a) and cooling (b) process and the thixotropic properties (c) of MAG-oleogel-based W/O HIPPEs (φ = 75% and 80%) before and after 4 freeze–thaw cycles; Figure S3: Appearance of GMS-oleogel-based W/O HIPPEs (φ = 85%) after 14-d, 30-d and 100-d storage; Figure S4: Effect of whipping on the short-term storage stability on the bread (a) and microstructure (b) of fresh oleogel-based W/O HIPPEs (φ = 85%) with different internal phase compositions.
