# Supplementary material for: Fabrication and Stability Improvement of Monoglyceride Oleogel/Polyglycerol Polyricinoleate-Stabilized W/O High Internal Phase Pickering Emulsions
Source: Foods. 2024 Jun 20;13(12):1944. doi: 10.3390/foods13121944 (PMC11203119; doi:10.3390/foods13121944)
Supplement: Supplementary file 1 [file foods-13-01944-s001.zip › foods-3055541-supplementary.pdf]

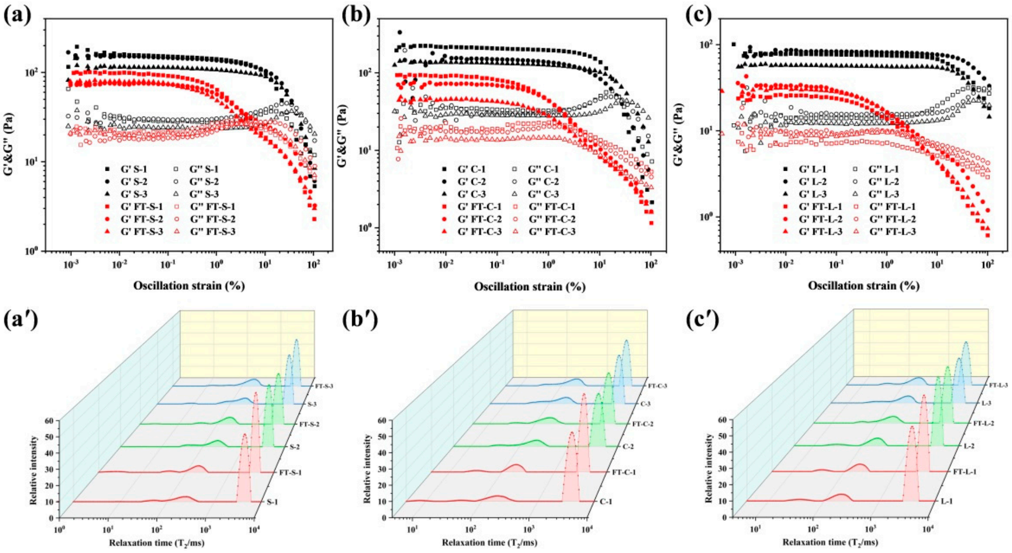

Figure S1. Strain sweep curves of GMS (**a**), GMC (**b**) and GML (**c**) oleogel-based W/O HIPPEs before and after freeze–thaw cycles. Water distribution of GMS (**a′**), GMC (**b′**) and GML (**c′**) oleogel-based W/O HIPPEs before and after freeze–thaw cycles.

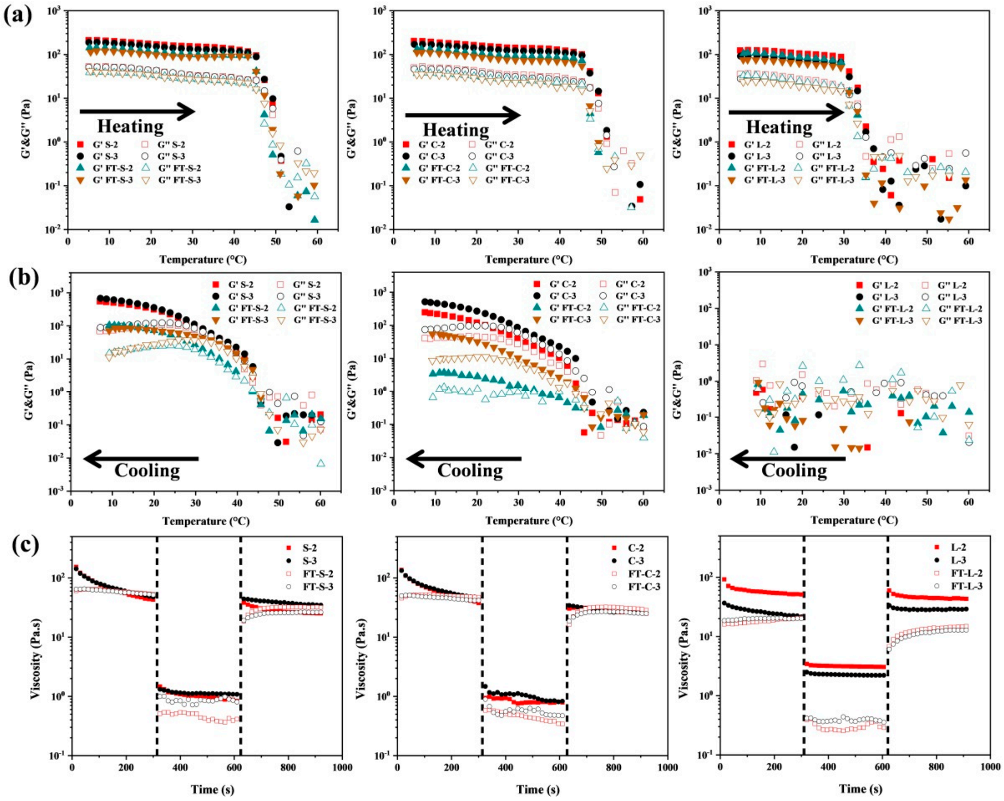

Figure S2. Temperature sweep curves during heating (**a**) and cooling (**b**) process and the thixotropic properties (**c**) of MAG-oleogel-based W/O HIPPEs (φ = 75% and 80%) before and after 4 freeze–thaw cycles.

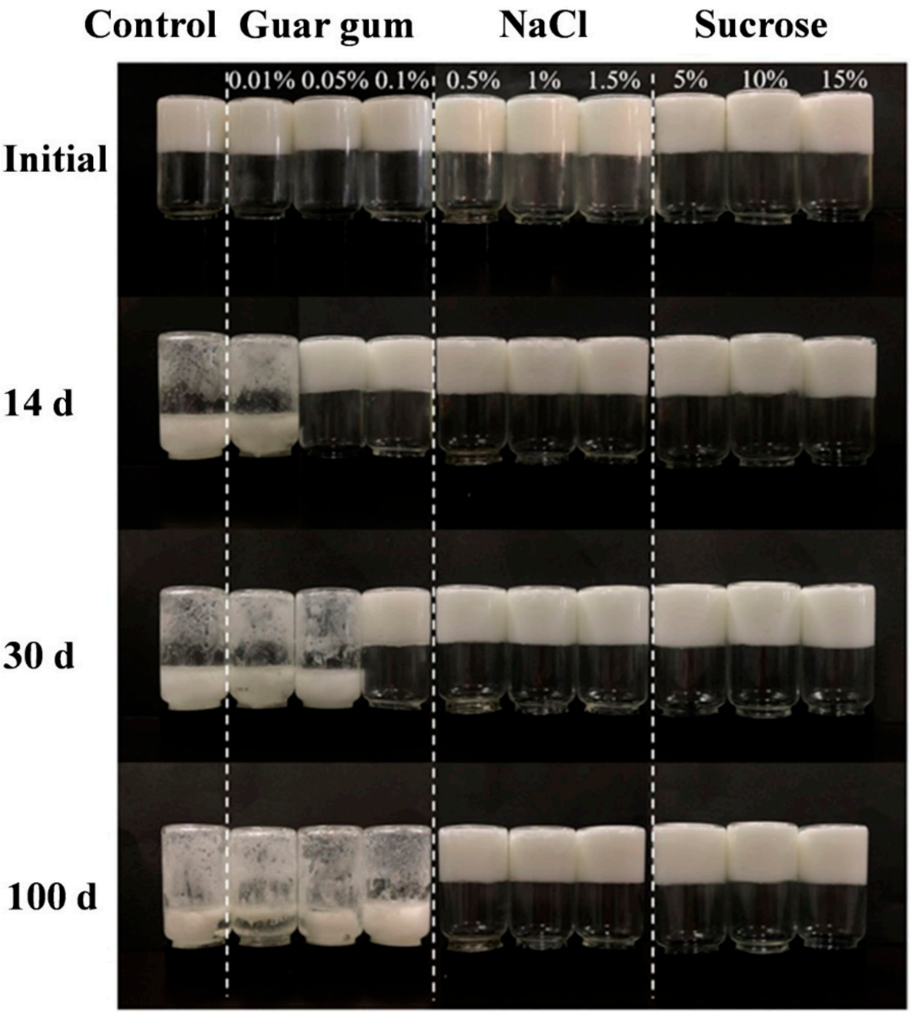

Figure S3. Appearance of GMS-oleogel-based W/O HIPPEs (φ = 85%) after 14-d, 30-d and 100-d storage.

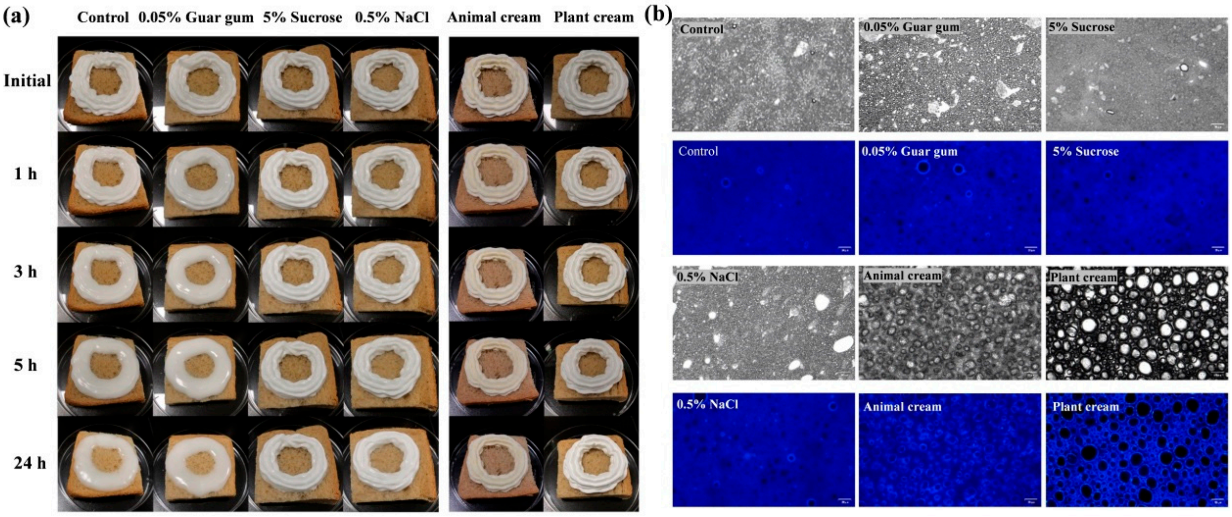

Figure S4. Effect of whipping on the short-term storage stability on the bread (**a**) and microstructure (**b**) of fresh oleogel-based W/O HIPPEs (φ = 85%) with different internal phase compositions.
